# MicroRNA-21a-5p inhibition alleviates systemic sclerosis by targeting STAT3 signaling

**DOI:** 10.1186/s12967-024-05056-3

**Published:** 2024-04-01

**Authors:** Jin-Sil Park, Chongtae Kim, JeongWon Choi, Ha Yeon Jeong, Young-Mee Moon, Hoin Kang, Eun Kyung Lee, Mi-La Cho, Sung-Hwan Park

**Affiliations:** 1 The Rheumatism Research Center, Catholic Research Institute of Medical Science, College of Medicine, The Catholic University of Korea, 222 Banpo-Daero, Seocho-gu, Seoul, 06591 South Korea; 2 Divison of Rheumatology, Department of Internal Medicine, Seoul St. Mary’s Hospital, The Catholic University of Korea, 222 Banpo-Daero, Seocho-Gu, Seoul, 06591 South Korea; 3 Department of Medical Lifescience, College of Medicine, The Catholic University of Korea, Seoul, South Korea; 4 Department of Biomedicine & Health Sciences, College of Medicine, The Catholic University of Korea, Seoul, South Korea; 5 Department of Biochemistry, College of Medicine, The Catholic University of Korea, 222 Banpo-Daero, Seocho-gu, Seoul, 06591 South Korea; 6 Lab of Translational ImmunoMedicine, Catholic Research Institute of Medical Science, College of Medicine, The Catholic University of Korea, 222 Banpo-Daero, Seocho-gu, Seoul, 06591 South Korea

## Abstract

**Background:**

MicroRNA (miRNA)-21-5p participates in various biological processes, including cancer and autoimmune diseases. However, its role in the development of fibrosis in the in vivo model of systemic sclerosis (SSc) has not been reported. This study investigated the effects of miRNA-21a-5p overexpression and inhibition on SSc fibrosis using a bleomycin-induced SSc mouse model.

**Methods:**

A murine SSc model was induced by subcutaneously injecting 100 μg bleomycin dissolved in 0.9% NaCl into C57BL/6 mice daily for 5 weeks. On days 14, 21, and 28 from the start of bleomycin injection, 100 μg pre-miRNA-21a-5p or anti-miRNA-21a-5p in 1 mL saline was hydrodynamically injected into the mice. Fibrosis analysis was conducted in lung and skin tissues of SSc mice using hematoxylin and eosin as well as Masson’s trichrome staining. Immunohistochemistry was used to examine the expression of inflammatory cytokines, phosphorylated signal transducer and activator of transcription-3 (STAT3) at Y705 or S727, and phosphatase and tensin homologue deleted on chromosome-10 (PTEN) in skin tissues of SSc mice.

**Results:**

MiRNA-21a-5p overexpression promoted lung fibrosis in bleomycin-induced SSc mice, inducing infiltration of cells expressing TNF-α, IL-1β, IL-6, or IL-17, along with STAT3 phosphorylated cells in the lesional skin. Conversely, anti-miRNA-21a-5p injection improved fibrosis in the lung and skin tissues of SSc mice, reducing the infiltration of cells secreting inflammatory cytokines in the skin tissue. In particular, it decreased STAT3-phosphorylated cell infiltration at Y705 and increased the infiltration of PTEN-expressing cells in the skin tissue of SSc mice.

**Conclusion:**

MiRNA-21a-5p promotes fibrosis in an in vivo murine SSc model, suggesting that its inhibition may be a therapeutic strategy for improving fibrosis in SSc.

## Introduction

Systemic sclerosis (SSc) or scleroderma is a complex autoimmune connective tissue disease marked by uncontrolled fibroblast activation [1]. Bairkdar and colleagues performed a meta-analysis and reported that the overall pooled prevalence of SSc was 17.6 per 100,000 and the overall pooled incidence rate of SSc was 1.4 per 100,000 person-year [2]. Although the prevalence of SSc is relatively low, SSc has the highest mortality rate among all rheumatic diseases, with life loss of up to 11.3 years for women and 25.8 years for men [3]. SSc affects skin and multiple internal organ such as heart, lung, and kidney, but there is still no curative treatment for SSc [4].

While its cause remains unknown, chronic inflammation triggers sustained myofibroblast activation, leading to excessive deposition of extracellular matrix components, including collagen and glycoproteins, resulting in fibrosis and impaired cellular function [5, 6]. Transforming growth factor-β (TGF-β) is a potent profibrotic factor in SSc fibrosis [7]. Mothers against decapentaplegic homologue (SMAD)2 and SMAD3, downstream of TGF-β, promote fibrosis, while SMAD7, a negative regulator, can protect against TGF-β-mediated fibrosis [8].

MicroRNAs (miRNAs) are small non-coding RNAs (approximately 20–22 nucleotides) that regulate gene expression post-transcriptionally [9]. Recent observations highlight their involvement in biological processes, including cell proliferation, differentiation, and apoptosis [10, 11]. Aberrant expression of miRNAs is closely linked to the pathophysiology of diseases, suggesting that circulating miRNAs are potential biomarkers and therapeutic targets [12]. Studies have reported an altered miRNA profile in the serum of SSc patients, with differentially expressed miRNAs implicated in various fibrotic processes [13–15], indicating a potential role for miRNAs in SSc development. Based on the study of the miRNA profile that changes in disease conditions, the development of therapeutics using the form of miRNA mimics or anti-miRs is considered a promising treatment strategy. Several miRNA-based therapeutics for human diseases have reached the clinical development stage [16, 17].

Among extensively studied miRNAs, miRNA-21-5p is involved in multiple biological processes, including cancer, immune responses, and autoimmune diseases [18, 19]. Previous studies have demonstrated its role in regulating skin fibrosis, with elevated levels in the serum and skin tissue of SSc patients [20]. MiRNA-21-5p induction through TGF-β in fibroblasts promotes fibrosis by suppressing and regulating SMAD7, a negative regulator of TGF-β [21]. However, there are no reports on the effects of miRNA-21 overexpression or inhibition on SSc fibrosis in in vivo systems.

The objective of this study is to determine the effect of miRNA-21-5p on fibrosis development in SSc. Using a bleomycin-induced murine SSc model, we demonstrated for the first time that inhibiting miRNA-21a-5p improved fibrosis development by lowering signal transducer and activator of transcription 3 (STAT3) and increasing phosphatase and tensin homologue deleted on chromosome-10 (PTEN) in an in vivo system. These results suggest that miRNA-21 is a promising therapeutic target for SSc.

## Materials and methods

### Animals and treatment

We obtained 8-week-old male C57BL/6 mice from Jackson Laboratory (Bar Harbor, ME, USA). To create a murine model of SSc, mice received daily subcutaneous injections of 50 µg of bleomycin (BLM, HY-17565A, MedChemExpress) dissolved in 100 µL phosphate-buffered saline (PBS; CBP3071, Dynebio) for 2 weeks. On days 14, 21, and 28 from the start of bleomycin injection, mice were hydrodynamically injected with 100 µg of plasmids containing pre-miRNA-21a-5p (#PMIRH21PA-1) [22], negative control (pre-miR-NC), anti-sense miR against miRNA-21a-5p (miRZip-21 lentiviral vector expressing anti-sense miRNA-21a-5p, anti-miRNA-21a-5p, #mZIP21-PA-1) [23] or miRZip-scrambled hairpin vector (scrambled anti-miR, #mZIP000-PA-1) (System Bioscience Inc) in 1 mL saline (n = 5/group). #PMIRH21PA-1 is an expression vector containing the miR-21 precursor construct and #mZIP21-PA-1 represses miR-21. Each plasmid was hydrodynamically injected intravenously into the tail vein. The mice were sacrificed on day 33 from the start of bleomycin injection, using 2.5% isoflurane inhalation, followed by histologic analysis. The mice were housed under specific-pathogen-free conditions at the Institute of Medical Science of the Catholic University of Korea and provided standard mouse diet and water. All experimental procedures were approved by the Animal Research Ethics Committee of the Catholic University of Korea, adhering to National Institutes of Health guidelines (Permit number: 2017–0067–02).

### Histology

The skin tissues of mice were fixed in 10% neutral-buffered formalin (HT501320, Sigma) and embedded in paraffin. Sections (5 µm thick) were stained with hematoxylin and eosin (H&E) and Masson’s trichrome (MT), as described previously [24]. MT staining was conducted using ready–to-use kit (Trichrome Stain (Masson) Kit, HT15, Sigma-Aldrich). After deparaffinization and rehydration, the lung section slides were immersed in Bouin’s solution (HT 10132, Sigma-Aldrich) at 56 °C for 15 min. Subsequently, the slides were washed with tap water for 5 min. Next, the tissues were stained in Weigert’s hematoxylin for 5 min, and then washed again with tap water for 5 min. Then, the slides were stained in Biebrich scarlet-acid fuchsin for 5 min, rinsed in distilled water, incubated in phosphotungstic-phosphomolybdic acid for 5 min, dyed with aniline blue for 5 min, and fixed in 1% acetic acid for 2 min. Finally, the slides were washed in distilled water, dehydrated and mounted.

### Histologic analysis

Dermal thickness and lung severity were scored as described previously [25–27]. Dermal thickness, which is the thickness of skin from the top of the granular layer to the junction between the dermis and subcutaneous fat, was measured. Fibrosis score was obtained by analyzing lung-cross section slides stained with H&E and MT. The extent of fibrotic lesions was scored as 0 (0–10%), 1 (10–25%), 2 (25–50%), 3 (50–75%), or 4 (75–100%). The severity of fibrosis was scored from 0 (normal lung) to 8 (total fibrosis), referring to the Ashcroft scoring system. The final fibrosis score was calculated by multiplying the extent of fibrotic lesions by the Ashcroft score [27].

### Immunohistochemistry

Following deparaffinization, tissue Sects. 5 µm thick were subjected to antigen retrieval using proteinase K (Dako, #S3020) in Tris–EDTA buffer (Sigma-Aldrich, #93,283). To inhibit endogenous peroxidase activity, the sections were treated with 3% hydrogen peroxide (Samchun Chemicals, #H0300) in methyl alcohol (Duksan, #59). Then they were incubated overnight at 4 °C with primary antibodies against tumor necrosis factor-α (TNF-α, Abcam, #Ab6671), interleukin (IL)-1β (NOVUS #NB600–633), IL-6 (NOVUS, #NB600–1131), IL-17 (Abcam, #ab79056), STAT3 (Abcam, #ab68153), phosphorylated STAT3 at tyrosine 705 (Y705) (Abcam, #ab76315) or serine 727 (S727) (Abcam, #ab30647), and PTEN (R&D Systems, #AF847). After 2 h incubation at room temperature with the above antibodies, the sections were incubated for 30 min with horseradish peroxidase-conjugated secondary antibody. The final product was developed using DAB chromogen (Dako). Positive cells, identified based on dark-brown deposits in lymphocyte nuclei, were counted in 10 randomly selected high-power fields.

### Statistical analysis

Data are expressed as mean ± standard deviation (± SD). Statistical significance was assessed using one-way analysis of variance (ANOVA) and Tukey’s test using GraphPad PRISM Version 5.0 (GraphPad Software, USA). Statistical significance was defined as *p* < 0.05.

## Results

### MiRNA-21a-5p accelerates lung fibrosis in the bleomycin-induced fibrosis model

**Fig. 1:**
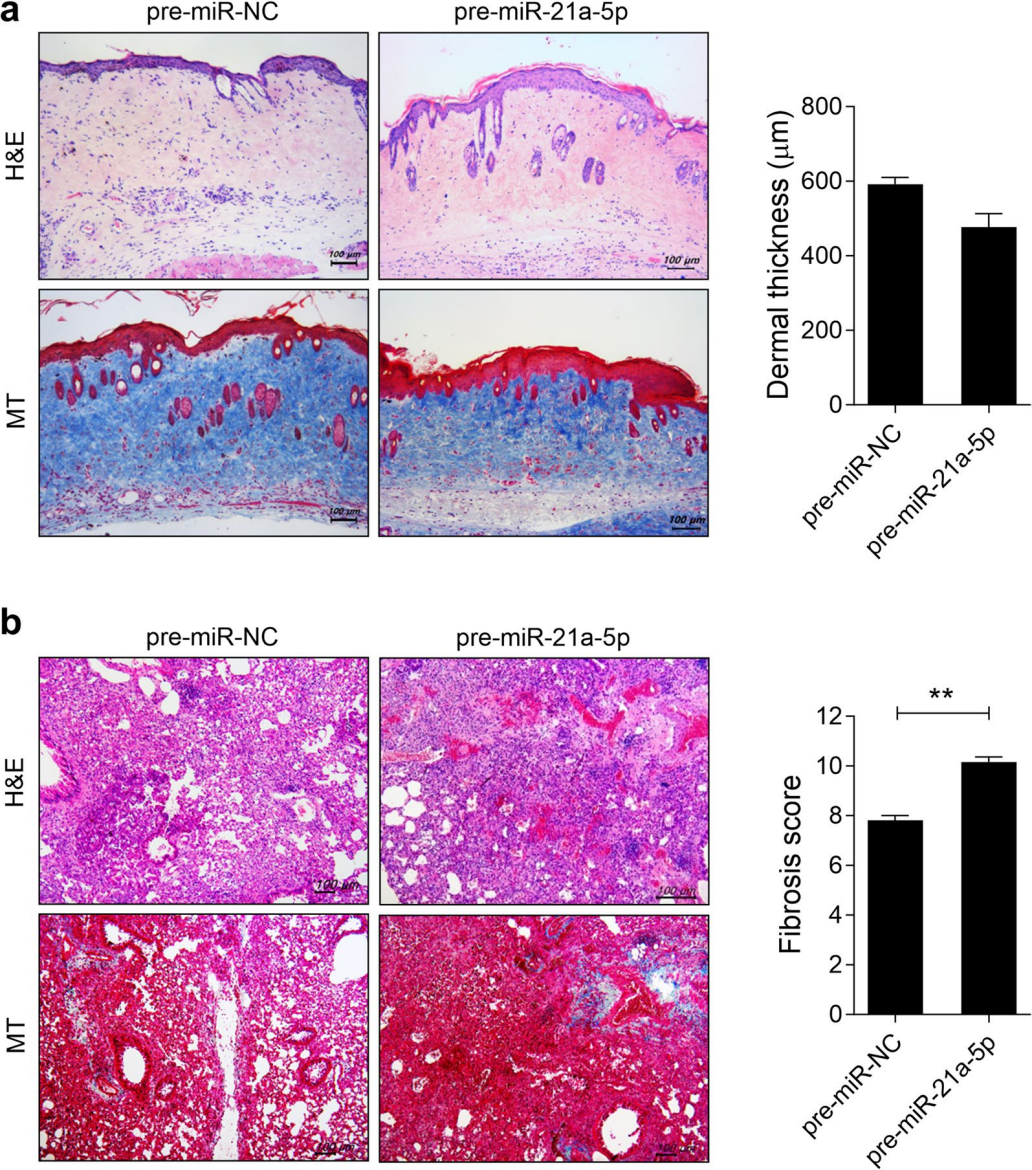
MiRNA-21a-5p overexpression promotes skin and lung fibrosis in bleomycin-induced SSc mice. The SSc murine model was induced by subcutaneously injecting 100 μg bleomycin dissolved in 100 μL PBS into 8 week-old C57BL/6 mice daily for 5 weeks. On days 14, 21, and 28 from the start of bleomycin injection, plasmid containing of 100 μg pre-miRNA-21a-5p (n = 5) or pre-miR-NC as a control (n = 5) in 1 mL saline was hydrodynamically injected into the mice. The mice were sacrificed on day 33 from the start of bleomycin injection, and histologic analysis was performed. **a**, **b** Representative images of the skin stained with hematoxylin and eosin and Masson’s trichrome (MT) **a** and lung **b** tissues of bleomycin-induced mice injected with plasmid containing of pre-miRNA-21a-5p or pre-miR-NC. The graphs show the skin dermal thickness **a** and pulmonary fibrosis score **b**. Original magnification: 100 × . Scale bar: 100 µm

To investigate the pathological impact of miRNA-21a-5p on skin and lung fibrosis in vivo, we assessed fibrosis development through hydrodynamic injection of plasmids containing pre-miRNA-21a-5p, which is an expression vector containing the miR-21 precursor construct, or negative control (pre-miR-NC) into a bleomycin-induced SSc murine model. On days 14, 21, and 28 from the start of bleomycin injection, mice were hydrodynamically injected with 100 µg of plasmids containing pre-miRNA-21a-5p or pre-miR-NC in 1 mL saline (n = 5/group). Treatment with pre-miRNA-21a-5p did not alter the thickness of the dermal skin compared to BLM-induced SSc mice injected with pre-miR-NC (Fig. 1). However, pre-miRNA-21a-5p treatment promoted fibrosis and collagen deposition in the lungs of BLM-induced SSc mice. These findings suggest that miRNA-21a-5p may function as a pathogenic factor, potentially accelerating SSc progression.

### MiRNA-21a-5p increases the number of inflammatory cytokine-secreting cells

**Fig. 2:**
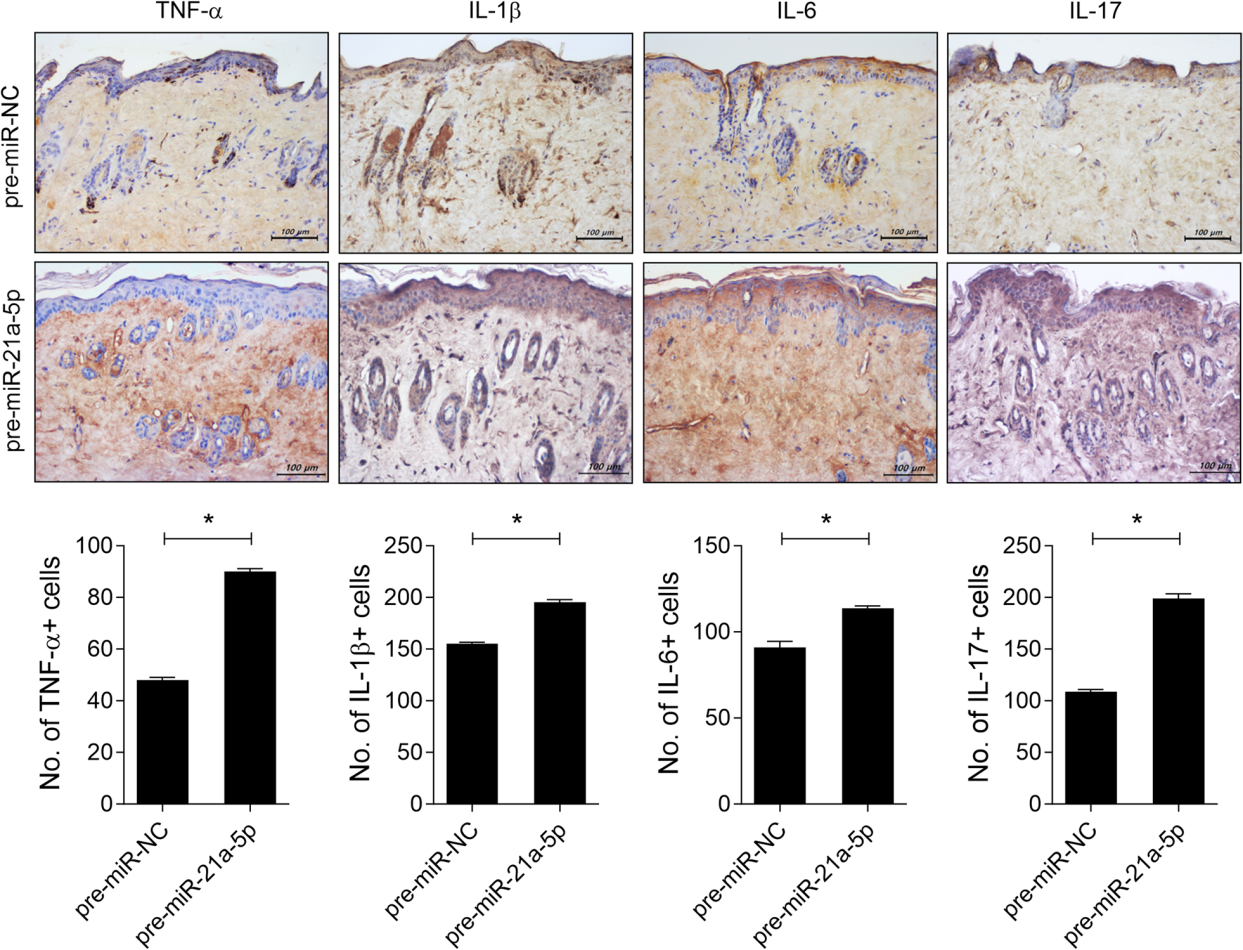
MiRNA-21a-5p treatment enhances inflammatory cell infiltration into the skin of bleomycin mice. On days 14, 21, and 28 from the start of bleomycin injection, plasmid containing of 100 μg pre-miRNA-21a-5p (n = 5) or pre-miR-NC as a control (n = 5) in 1 mL saline was hydrodynamically injected into the mice. The mice were sacrificed on day 33 from the start of bleomycin injection, and immunohistochemistry was performed on the skin tissue. Sections of skin tissue were stained with antibodies against TNF-α, IL-β, IL-6, and IL-17. The graphs present the number of antibody-positive cells (mean ± SD, n = 5/group). Original magnification: 200 × . Scale bar: 100 µm

**Fig. 3:**
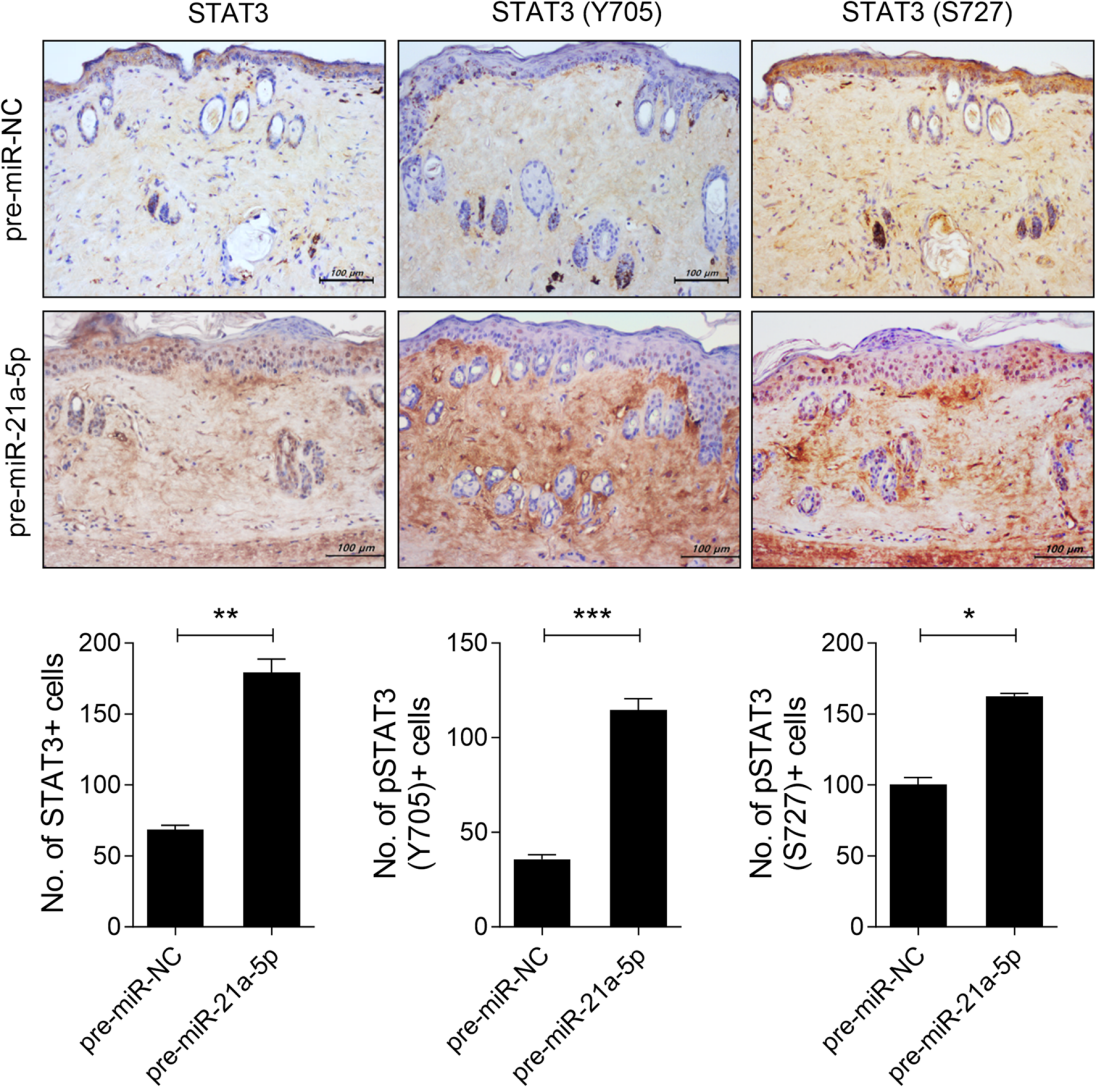
MiRNA-21a-5p increases the number of STAT3-phosphorylated cells in the skin of bleomycin mice. On days 14, 21, and 28 from the start of bleomycin injection, plasmid containing of pre-miRNA-21a-5p (n = 5) or pre-miR-NC as a control (n = 5) was hydrodynamically injected into the mice. The mice were sacrificed on day 33 from the start of bleomycin injection, and skin tissue sections were stained with antibodies against STAT3, phosphorylated STAT3 at Y705, or S727. The graphs present the number of antibody-positive cells (mean ± SD, n = 5/group). Original magnification: 200 × . Scale bar: 100 µm

To assess the impact of miRNA-21a-5p on the infiltration of inflammatory cells in the skin of SSc mice, we conducted immunohistochemistry on the skin tissue of BLM-induced SSc mice injected with either plasmid containing pre-miRNA-21a-5p or pre-miR-NC. MiRNA-21a-5p overexpression led to increased infiltration of cells producing TNF-α, IL-1β, IL-6, or IL-17 compared to the control group (Fig. 2). These cytokines activate the STAT3 pathway [28–30], which then promote fibroblast activation and tissue fibrosis [31]. We further investigated the role of miRNA-21a-5p in STAT3 phosphorylation in the skin of SSc mice. Treatment of plasmid containing miRNA-21a-5p increased the number of STAT3-positive cells in the skin tissue of SSc mice, particularly those with STAT3 phosphorylation at tyrosine 705 and serine 727 compared to the control group (Fig. 3). These findings suggest that miRNA-21a-5p may contribute to tissue fibrosis in SSc by activating cells that secrete various inflammatory cytokines and promoting STAT3 phosphorylation in the skin tissue.

### MiRNA-21a-5p inhibition attenuates bleomycin-induced dermal fibrosis in mice

**Fig. 4:**
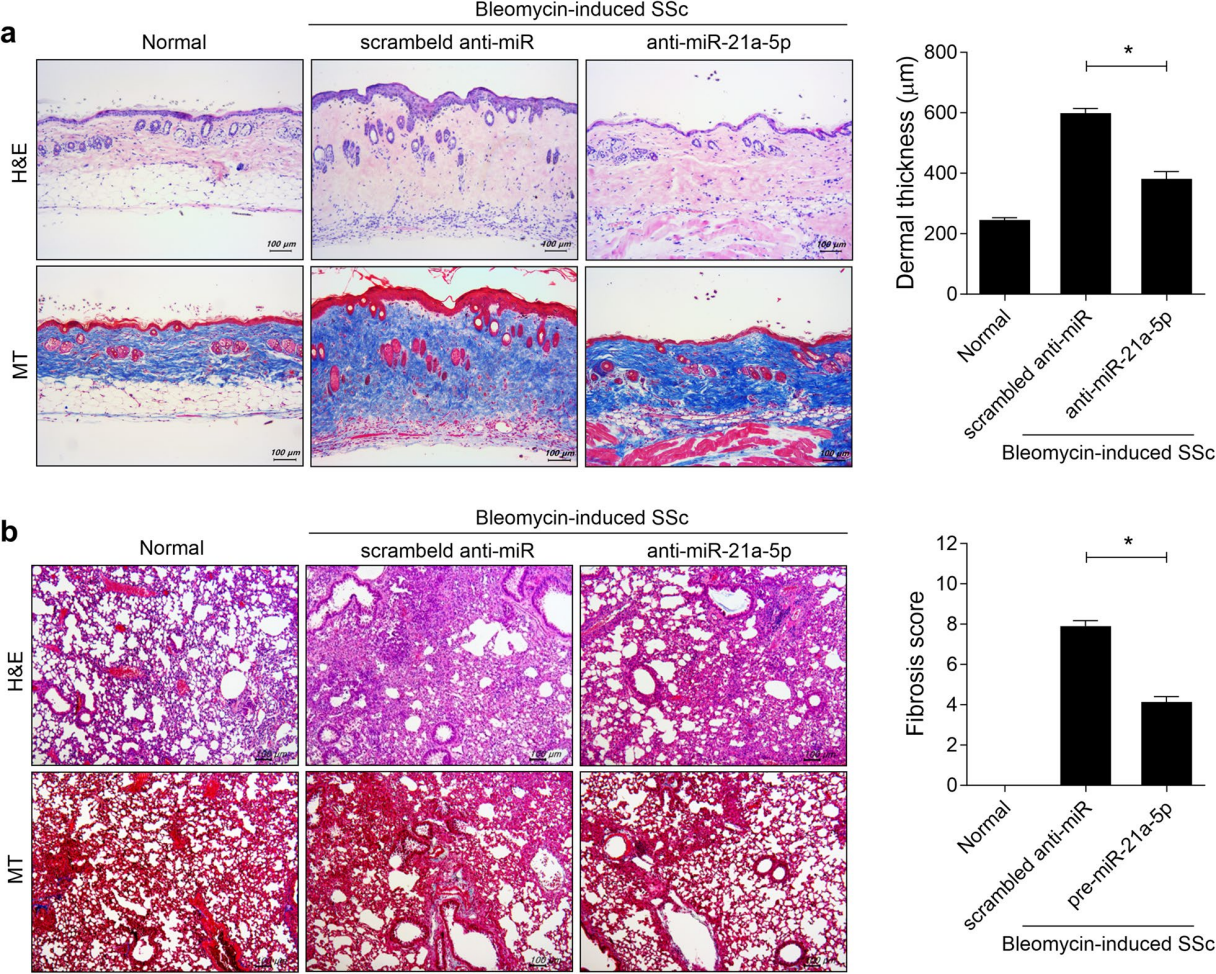
MiRNA-21a-5p inhibition promotes skin and lung fibrosis in bleomycin-induced SSc mice. On days 14, 21, and 28 from the start of bleomycin injection, 100 μg miRZip-scrambled hairpin vector (scrambled anti-miR) as a control (n = 5) or anti-sense miR against miRNA-21a-5p (anti-miRNA-21a-5p), which inhibits miR-21a-5p (n = 5) in 1 mL saline was hydrodynamically injected into the mice. The mice were sacrificed on day 33 from the start of bleomycin injection, and histologic analysis was performed. **a**, **b** Representative images of the skin stained with hematoxylin and eosin and Masson’s trichrome (MT) **a** and lung tissue **b** of bleomycin-induced mice injected with scrambled anti-miR or anti-miRNA-21a-5p. The graphs show the skin dermal thickness **a** and pulmonary fibrosis score **b**. Original magnification: 100 × . Scale bar: 100 µm

To evaluate the impact of miRNA-21a-5p blockade on skin fibrosis in vivo, we compared the bleomycin-induced scleroderma model treated with plasmid containing of anti-sense miR against miRNA-21a-5p (miRZip-21 lentiviral vector expressing anti-sense miRNA-21a-5p, anti-miRNA-21) to that treated with miRZip-scrambled hairpin vector (scrambled anti-miR). On days 14, 21, and 28 from the start of bleomycin injection, mice were hydrodynamically injected with 100 µg of anti-miRNA-21a-5p or scrambled anti-miR in 1 mL saline (n = 5/group). Hydrodynamic injection of anti-miRNA-21a-5p resulted in reduced skin dermal thickness and alleviated collagen deposition compared to the group injected with scrambled anti-miR (Fig. 4). Moreover, miRNA-21a-5p inhibition led to an improvement in fibrosis development and collagen deposition in the lungs of BLM-induced SSc mice. These findings suggest that miRNA-21a-5p blockade can mitigate SSc progression.

### MiRNA-21a-5p suppression reduces infiltration of cells secreting inflammatory cytokines in lesional skin

**Fig. 5:**
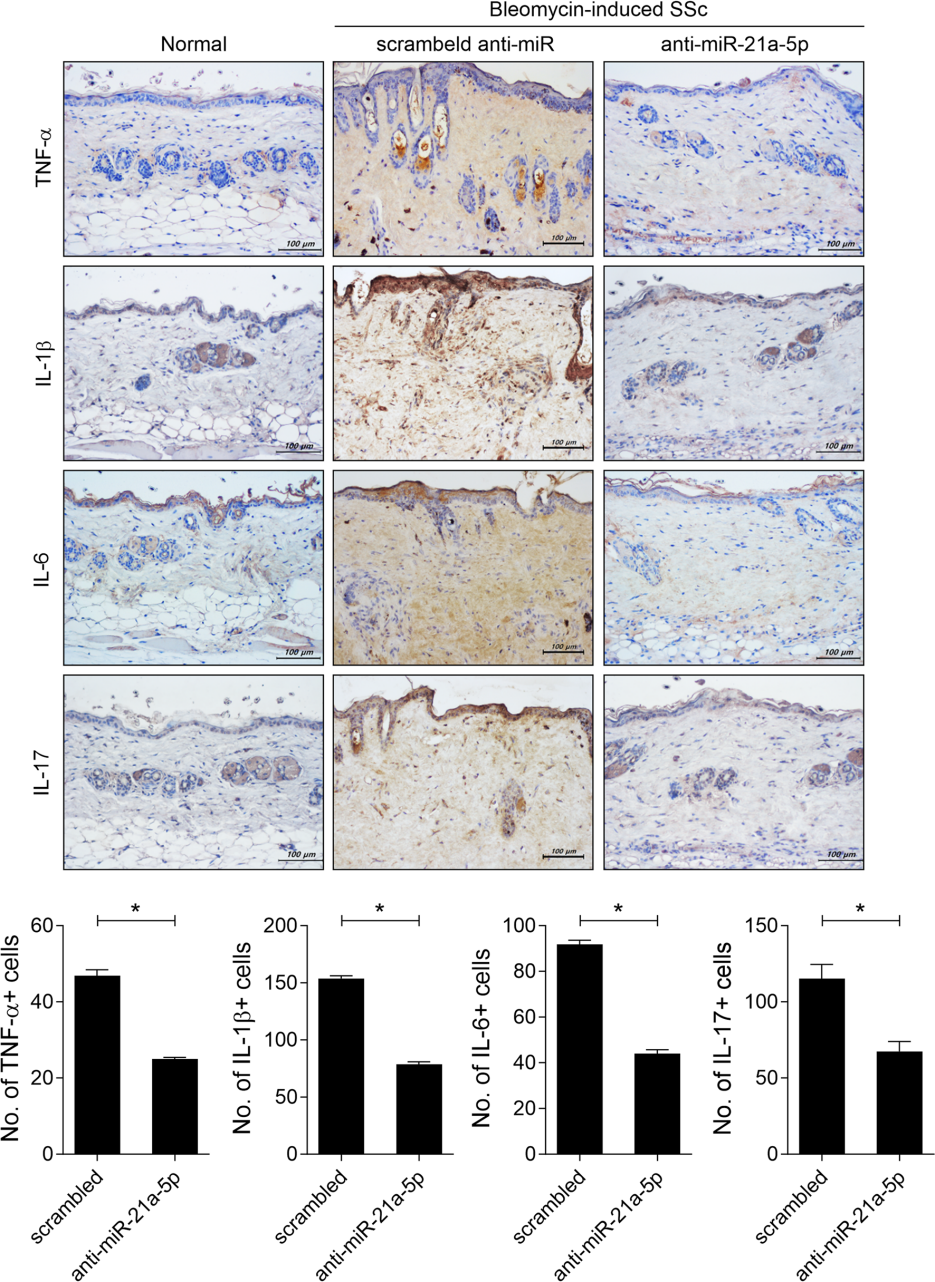
MiRNA-21a-5p inhibition decreases inflammatory cell infiltration into the lesional skin of bleomycin-induced SSc mice. On days 14, 21, and 28 from the start of bleomycin injection, plasmid containing of 100 μg scrambled anti-miR (n = 5) or anti-miRNA-21a-5p (n = 5) in 1 mL saline was hydrodynamically injected into the mice. The mice were sacrificed on day 33 from the start of bleomycin injection, and skin tissue sections were immunohistochemically stained for TNF-α, IL-β, IL-6, and IL-17. The graphs present the number of antibody-positive cells (mean ± SD, n = 5/group). Original magnification: 200 × . Scale bar: 100 µm

To determine whether miRNA-21a-5p inhibition could reduce the infiltration of cells secreting inflammatory cytokines in the lesional skin of SSc mice, we investigated the expression of inflammatory cytokines in the skin tissue of BLM-induced SSc mice treated with anti-miRNA-21. MiRNA-21a-5p inhibition through plasmid containing of anti-miRNA-21a-5p treatment lowered the infiltration of cells secreting TNF-α, IL-β, IL-6, or IL-17 in the lesional skin tissue of SSc mice (Fig. 5).

### MiRNA-21a-5p inhibition reduces STAT3-phosphorylated cell invasion and increases the number of PTEN-positive cells in the skin tissue of BLM-induced SSc mice

**Fig. 6:**
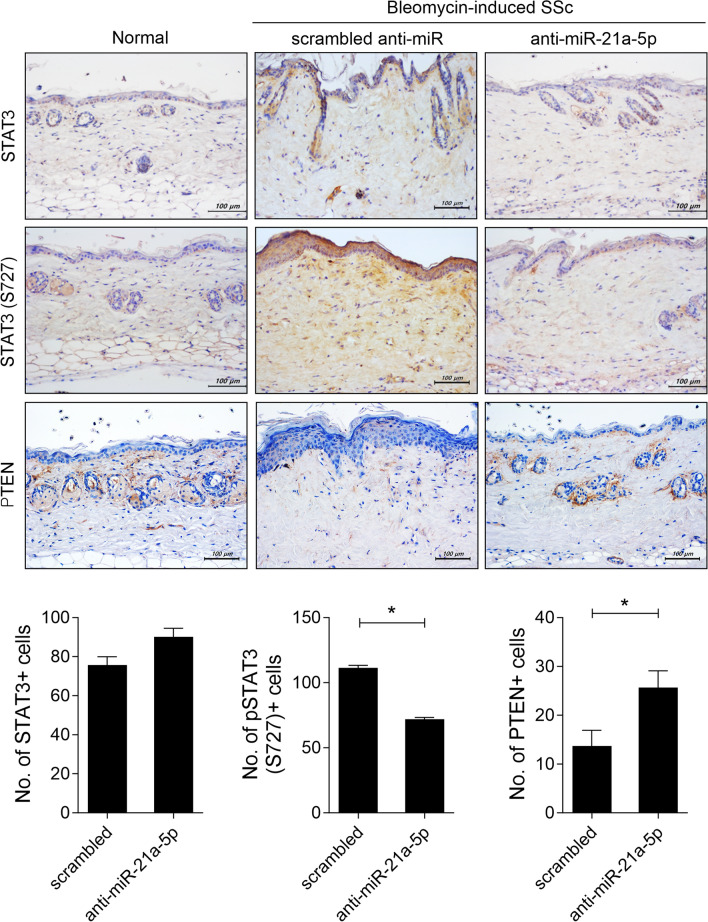
MiRNA-21a-5p inhibition prevented STAT3-phosphorylated cell invasion and increased the number of PTEN-positive cells in the skin tissue of bleomycin-induced SSc mice. On days 14, 21, and 28 from the start of bleomycin injection, plasmid containing of scrambled anti-miR (n = 5) or anti-miRNA-21a-5p (n = 5) was hydrodynamically injected into the mice. The mice were sacrificed on day 33 from the start of bleomycin injection, and skin tissue sections were stained with antibodies against STAT3, phosphorylated STAT3 at S727, and PTEN. The graphs present the number of antibody-positive cells (mean ± SD, n = 5/group). Original magnification: 200 × . Scale bar: 100 µm

Subsequently, we examined whether blocking miRNA-21a-5p affected phosphorylated STAT3-positive cell infiltration within lesional skin in BLM-induced SSc mice. Compared to the control group injected with scrambled anti-miR, there were no differences in the infiltration of STAT3-positive cells. However, the number of phosphorylated STAT3-positive cells was significantly reduced in the skin tissue of mice injected with anti-miRNA-21a-5p (Fig. 6). Previous studies have reported that PTEN knockout in fibroblasts increases collagen deposition [32], and PTEN levels are low, with an expression negatively correlated with the miRNA-21-5p level in human keloid fibroblasts [33]. Therefore, we investigated whether miRNA-21a-5p inhibition affects PTEN level in the skin of SSc mice. Anti-miRNA-21a-5p treatment increased PTEN-positive cell infiltration into the lesional skin of SSc mice (Fig. 6). These results suggest that controlling the action of miRNA-21-5p may lead to SSc improvement.

## Discussion

The present study demonstrated that miRNA-21a-5p inhibition ameliorated fibrosis by reducing phosphorylated STAT3-positive cell infiltration and increasing PTEN-positive cell infiltration in the lesional skin of SSc mice. In the skin of BLM-induced SSc mice, miRNA-21a-5p overexpression increased the infiltration of cells expressing inflammatory factors, including TNF-α, IL-1β, IL-6, and IL-17, as well as STAT3 phosphorylated cells. Conversely, miRNA-21a-5p inhibition through plasmid containing anti-miRNA-21a-5p treatment reduced the number of inflammatory cytokine-positive cells in the skin tissue of BLM-induced SSc mice. Moreover, miRNA-21a-5p inhibition decreased STAT3-phosphorylated cell infiltration and increased the infiltration of cells positive for PTEN, a regulator of collagen deposition [32], in the skin tissue of SSc mice. Based on the elevated miRNA-21a-5p expression in the SSc mouse model and its reported promotion of fibrogenic skin activation [21], our findings suggest that blocking miRNA-21-5p could be a therapeutic strategy for alleviating SSc development.

MiRNAs play a pivotal role in the pathogenesis of tissue fibrosis in SSc, influencing ECM synthesis and TGF-β signaling [9]. In particular, dysregulated miRNA expression associated with SSc fibrosis has been reported in the skin tissue of diffuse cutaneous SSc patients. These miRNAs regulate the expression of fibrosis-related target genes, including SMAD3 and SMAD7 [34]. Among the significantly increased miRNAs in SSc, miRNA-21-5p has been extensively investigated for its potential role in skin fibrosis. Elevated miRNA-21-5p levels have been reported in the serum of SSc patients [20], and TGF-β stimulation in skin fibroblasts increases miRNA-21-5p, which negatively regulates SMAD7, exhibiting profibrogenic effects [21]. MiRNA-21-5p overexpression in SSc fibroblasts increases the mRNA and protein levels of the anti-apoptotic gene Bcl2, while inhibition of miRNA-21-5p induces apoptosis in apoptosis-resistant SSc fibroblasts [35]. Despite substantial evidence suggesting a pathogenic role for miRNA-21-5p in progressive fibrosis in SSc, the efficacy of miRNA-21-5p has not been investigated in an in vivo system of SSc. The present study demonstrated that miRNA-21a-5p overexpression promotes inflammatory cell infiltration and exacerbates fibrosis in lesional skin of BLM-induced SSc mice. Conversely, miRNA-211-5p inhibition through plasmid containing anti-miRNA-21a-5p injection alleviated fibrosis in both lungs and skin, reducing inflammatory cell infiltration in skin tissue. In particular, anti-miRNA-21a-5p plasmid treatment increased PTEN expression, which negatively regulates collagen deposition in the lesional skin [32]. TGF-β upregulates miRNA-21-5p and decreases PTEN expression in fibroblasts, and miRNA-21-5p inhibition restores the decreased PTEN expression caused by TGF-β [36, 37]. Although the molecular mechanism by which miRNA-21a-5p inhibition affects PTEN expression requires further investigation, our findings suggest that increased PTEN through miRNA-21a-5p inhibition may improve fibrosis by inhibiting collagen deposition.

STAT3 serves as a central integrator of TGF-β-mediated profibrotic effects. Increased STAT3 phosphorylation is observed in dermal fibroblasts of SSc patients, and TGF-β stimulation significantly increases STAT3 phosphorylation in healthy dermal fibroblasts [38, 39]. STAT3 inhibition through JAK2 inhibition or small molecule inhibitors reduces the activation of dermal fibroblasts and increase in collagen levels induced by TGF-β [38, 39]. TGF-β-mediated STAT3 phosphorylation is influenced not only by JAK2, but also by JNK, SRC, and c-ABL kinases in fibroblasts. Fibroblast-specific knockout of STAT3 alleviates the development of bleomycin-induced skin fibrosis [31]. The profibrotic cytokine IL-6 induces collagen-1 synthesis by STAT3-dependent TGF-β-Smad3 activation through Gremlin, a bone morphogenetic protein antagonist [40]. Previous studies have demonstrated that miRNA-21-5p overexpression promotes cardiac fibrosis through STAT3 signaling [41] while STAT3 directly activates transcription of miRNA-21-5p, and increased miRNA-21-5p expression suppresses PTEN during cellular transformation [42]. Our findings demonstrate that miRNA-21a-5p overexpression promotes STAT3 phosphorylation in the skin tissues of SSc mice, while miRNA-21a-5p inhibition downregulates STAT3 phosphorylation. Although no studies have investigated the relationship between miRNA-21a-5p and STAT3 in lesional skin of SSc, our findings suggest that miRNA-21a-5p may act on STAT3-mediated fibrosis in SSc.

Our results were obtained by examining the levels of inflammation-related factors in the skin of mice that overexpressed or suppressed miR-21a-5p using immunohistochemistry. There is a need to confirm the results using various experimental techniques. To clarify the potential therapeutic target of miR-21 in SSc, further studies are needed to determine the mechanism of action of miR-21 in target cells. To this end, it is necessary to analyze cellular mRNA profile and cell phenotypic changes to determine in specific cells. To gain more insight, it is also necessary to investigate related signaling pathways by performing multi-omics analysis on specific cells in the skin of mice that overexpress or suppress miR-21a-5p.

## Conclusion

Our findings demonstrate that miRNA-21a-5p exacerbates fibrosis by promoting STAT3 phosphorylation and the expression of inflammation-related factors in an in vivo SSc mouse model. Furthermore, this was the first study to demonstrate that miRNA-21a-5p inhibition controls SSc fibrosis through STAT3 inhibition and PTEN upregulation. These findings highlight the potential of targeting miRNA-21 as a therapeutic strategy to alleviate SSc development.

## Data Availability

All data are available in the manuscript or upon request to the authors.
